# Mentorship of Junior Surgical Faculty Across Academic Programs in Surgery

**DOI:** 10.1001/jamasurg.2024.3390

**Published:** 2024-09-04

**Authors:** Jingjing Yu, Perisa Ruhi-Williams, Christian de Virgilio, Shahrzad Bazargan-Hejazi, Helen E. Ovsepyan, Steven D. Wexner, Katharine A. Kirby, Fatemeh Tajik, Angelina Lo, Aya Fattah, Farin F. Amersi, Kristine E. Calhoun, Lisa A. Cunningham, Paula I. Denoya, Henry R. Govekar, Sara M. Grossi, Jukes P. Namm, V. Prasad Poola, Robyn E. Richmond, Christine H. Rohde, Mayank Roy, Tara A. Russell, Nicola Sequeira, Anaar E. Siletz, Tiffany N. Tanner, Brian T. Valerian, Maheswari Senthil

**Affiliations:** 1 Department of Surgery, University of California Irvine Medical Center, Orange; 2 Department of Surgery, Harbor-UCLA Medical Center, Torrance, California; 3 Department of Psychiatry, Charles R. Drew University of Medicine and Science, Los Angeles, California; 4 Department of Preventative and Social Medicine, Charles R. Drew University of Medicine and Science, Los Angeles, California; 5 Department of Surgery, Cleveland Clinic Florida, Weston; 6 Center of Statistical Consulting, Department of Statistics, University of California Irvine, Irvine; 7 Department of Surgery, Cedars-Sinai Medical Center, Los Angeles, California; 8 Department of Surgery, University of Washington, Seattle; 9 Department of Surgery, The Ohio State University Medical Center, Columbus; 10 Department of Surgery, Stony Brook University, Stony Brook, New York; 11 Department of Surgery, Rush University Medical Center, Chicago, Illinois; 12 Department of Surgery, University of California San Diego, San Diego; 13 Department of Surgery, Loma Linda University, Loma Linda, California; 14 Departmeny of Surgery, Southern Illinois School of Medicine, Springfield; 15 Department of Surgery, Texas Tech University Health Sciences Center, Lubbock; 16 Department of Surgery, Columbia University Medical Center, New York, New York; 17 Department of Surgery, University of California Los Angeles, Los Angeles; 18 Department of Surgery, University of California Davis, Davis; 19 Department of Surgery, University of Southern California, Los Angeles; 20 Department of Surgery, University of Nebraska Medical Center, Omaha; 21 Department of Surgery, Albany Medical Center, Albany, New York

## Abstract

**Importance:**

Because mentorship is critical for professional development and career advancement, it is essential to examine the status of mentorship and identify challenges that junior surgical faculty (assistant and associate professors) face obtaining effective mentorship.

**Objective:**

To evaluate the mentorship experience for junior surgical faculty and highlight areas for improvement.

**Design, Setting, and Participants:**

This qualitative study was an explanatory sequential mixed-methods study including an anonymous survey on mentorship followed by semistructured interviews to expand on survey findings. Junior surgical faculty from 18 US academic surgery programs were included in the anonymous survey and interviews. Survey responses between “formal” (assigned by the department) vs “informal” (sought out by the faculty) mentors and male vs female junior faculty were compared using χ^2^ tests. Interview responses were analyzed for themes until thematic saturation was achieved. Survey responses were collected from November 2022 to August 2023, and interviews conducted from July to December 2023.

**Exposure:**

Mentorship from formal and/or informal mentors.

**Main Outcomes and Measures:**

Survey gauged the availability and satisfaction with formal and informal mentorship; interviews assessed broad themes regarding mentorship.

**Results:**

Of 825 survey recipients, 333 (40.4%) responded; 155 (51.7%) were male and 134 (44.6%) female. Nearly all respondents (319 [95.8%]) agreed or strongly agreed that mentorship is important to their surgical career, especially for professional networking (309 respondents [92.8%]), career advancement (301 [90.4%]), and research (294 [88.3%]). However, only 58 respondents (18.3%) had a formal mentor. More female than male faculty had informal mentors (123 [91.8%] vs 123 [79.4%]; *P* = .003). Overall satisfaction was higher with informal mentorship than formal mentorship (221 [85.0%] vs 40 [69.0%]; *P* = .01). Most male and female faculty reported no preferences in gender or race and ethnicity for their mentors. When asked if they had good mentor options if they wanted to change mentors, 141 (47.8%) responded no. From the interviews (n = 20), 6 themes were identified, including absence of mentorship infrastructure, preferred mentor characteristics, and optimizing mentorship.

**Conclusions and Relevance:**

Academic junior surgical faculty agree mentorship is vital to their careers. However, this study found that few had formal mentors and almost half need more satisfactory options if they want to change mentors. Academic surgical programs should adopt a framework for facilitating mentorship and optimize mentor-mentee relationships through alignment of mentor-mentee goals and needs.

## Key Points

**Question:**

What is the current mentorship experience for junior surgical faculty and what are the challenges to receiving effective mentorship?

**Findings:**

This qualitative study found that 18.3% of survey respondents had a formal mentor compared with 84.2% who had an informal mentor. Overall satisfaction with informal mentorship was higher than with formal mentorship, but research was the least helpful area for informal mentorship.

**Meaning:**

Mentorship is critical to career development in academic medicine, so improvement in mentor-mentee pairings and research mentorship is needed.

## Teaser

This qualitative study uses survey data to gauge the mentorship experience of junior surgical faculty and highlight areas for improvement.

## Introduction

Mentorship is a vital component of success across various disciplines, and academic surgery is no exception.^1,2,3^ Mentorship is critical for professional development and career advancement in surgery, especially for junior faculty during their formative years. Numerous studies have shown that mentorship can positively influence the career trajectory of junior faculty as it helps them navigate challenges, provides access to professional networks, increases scholarly productivity, and leads to higher rates of promotion.^4,5,6,7,8,9,10^

Despite the importance of mentorship in academic medicine,^11,12^ many gaps exist. Commonly reported concerns include the availability of suitable mentors, support for mentorship programs, and alignment of mentorship program goals with the mentees’ needs.^6,13,14^ A systematic review of mentoring in academic medicine revealed a surprisingly low percentage (20%) of faculty had mentors in some medical fields.^15^ In a 2016 study by Kibbe et al,^16^ only half (54%) of the 76 surgery program chairs reported having a mentorship program, and their structures varied widely. However, previous experience has shown that having a mentorship program alone may not be adequate for junior faculty success.^17^ Outcomes of a mandatory mentorship pilot program in a surgical department failed to show improvement in academic productivity, work-life integration, and overall job satisfaction.^17^ Additionally, significant gender disparities in mentorship exist. Sambunjak et al^15^ reported women perceived higher difficulty in finding mentorship than men. Shen et al^18^ also reported that women had less access to mentors, lower research productivity, and greater barriers to promotion.

Because of the prevailing widespread concerns with mentorship in academic medicine and the possibility that some of the challenges may be specific to surgery, we sought to evaluate the availability and scope of mentorship, as well as the challenges to obtaining effective mentorship for academic junior surgical faculty. In this study, Mentorship Across Academic Programs in Surgery (MAAPS), we aimed to (1) understand the mentees’ perceptions of mentorship, (2) identify differences in formal vs informal mentorship experiences among male and female junior surgical faculty, and (3) inform areas for improving surgical mentorship. We hypothesized that there is significant variability in mentorship structure and satisfaction between formal vs informal mentorship and that these differences may be influenced by gender.

## Methods

Our study used a 2-phase explanatory sequential mixed-methods design.^19^ In the first phase, we analyzed data from surveys on mentorship distributed to junior surgical faculty. In the second phase, we performed semistructured interviews with questions informed by the quantitative results to expand on the survey findings and further explore the mentorship experience.

### Participant Selection

We recruited 18 academic surgery programs across 5 US regions (West, Southwest, Midwest, Northeast, Southeast). A representative or “study champion” was designated at each institution to be responsible for obtaining study approval from the institutional review board and coordinating and promoting the study at their respective institutions.

Participants for both the survey and the semistructured interviews were junior surgical faculty (assistant or associate professor ranks). Participants had either a formal or informal mentor, both formal and informal mentors or neither. A “formal” mentor was assigned by the department while an “informal” mentor was sought out by the faculty. The study was approved by the institutional review board of each participating institution where required.

### Phase 1: Study Survey and Analysis Plan

Designed collaboratively by 5 surgeons (J.Y., P.R., C.d.V, S.B., M.S.), the self-administered anonymous survey comprised 26 items covering 4 primary areas: general mentorship experience, experience with formal mentorship, experience with informal mentorship, and demographic details (eAppendix 1 in Supplement 1). For questions assessing satisfaction, comfort, and agreement with an item, a 5-point Likert scale was used. The scale ranged from 1 (high dissatisfaction, discomfort, or disagreement) to 5 (high satisfaction, comfort, or agreement). Once developed, the survey was distributed to selected study champions to evaluate clarity, flow, and appropriateness of the questions. Based on the feedback, appropriate changes were made, and further validation was performed.

Through the online tool SurveyMonkey, a unique institutional survey link was sent to each study champion. The study champions then distributed the survey link to junior faculty at their respective institutions and encouraged participation by sending a minimum of 2 follow-up emails 1 month apart to reach a goal response rate of 50%. Participation in the survey was both anonymous and voluntary. Responses were collected between November 2022 and August 2023.

Demographic information was self-identified. Descriptive analysis was used to present an overview of overall sample characteristics and response rates. The scores 1/2 and 4/5 on the Likert scale were combined in the analysis. Bivariate analysis using χ^2^ tests was used for subgroup analysis comparing responses between male vs female faculty and faculty with formal vs informal mentors. The significance level was set at *P* < .05. Statistical analysis was completed using Stata version 18 (StataCorp).

### Phase 2: In-Depth Interview Design, Participant Selection, and Thematic Analysis

The second phase of the study involved semistructured interviews with junior surgical faculty who participated in the survey. Interview participants were selected using a nonprobability, snowball, purposive sampling method,^20^ allowing for the identification of potential interviewees through recommendations from initial participants. Verbal consent was obtained from each interview participant.

A semistructured interview guide was developed with questions designed based on the initial survey results to expand on our key findings (eAppendix 2 in Supplement 1). Interviews were conducted via an online video conferencing platform (Zoom) from July to December 2023 and took 15 to 30 minutes each. The interviews were conducted by a trained student researcher (A.F.). All interviews were systematically recorded and transcribed verbatim; then recordings were deleted. All names were redacted.

Interviews were analyzed to identify recurring themes in an inductive fashion. Steps for thematic analysis included familiarization with the data, selection of key quotes to reflect recurring patterns, development of codes based on keywords, and formation of themes after grouping keywords and corresponding coded data.^21,22^ The transcripts were independently coded by 2 coders (P.R. and M.S.), and coding disparities were discussed until consensus was reached.^22^ Interviews were conducted until thematic saturation was achieved. A total of 20 interviews were conducted (11 female and 9 male junior faculty).

## Results

### Phase 1: Survey Results

#### Study Participant Characteristics

Of 825 survey recipients, 333 (40.4%) responded. Self-reported demographic data of the survey participants are described in Table 1. One hundred fifty-five (51.7%) of the respondents self-identified as male and 134 (44.6%) as female. Most survey participants were aged 30 to 49 years (30-39 years: 115 [38.3%]; 40-49 years: 133 [44.3%]), White (187 [63.0%]), and assistant professors (172 [58.7%]) (Table 1). Of the junior faculty who responded, 58 (18.3%) had a formal mentor, 261 (84.2%) had at least 1 informal mentor, 47 (14.8%) had both formal and informal mentors, and 39 (12.3%) had no formal or informal mentors.

**Table 1.** Demographic Data for Junior Surgical Faculty Survey Participants

Characteristic	No. (%)
Gender	
Female	134 (44.6)
Male	155 (51.7)
Nonbinary/third gender	0
Prefer to self-describe	0
Prefer not to say	11 (3.7)
Other	0
Age, y	
30-39	115 (38.3)
40-49	133 (44.3)
50-59	36 (12.0)
≥60	5 (1.7)
Prefer not to say	11 (3.7)
Race and ethnicity (choose any)	
African	3 (1.0)
African American/Black	11 (3.7)
Asian American	34 (11.4)
East Asian	10 (3.4)
Hispanic/Latinx	22 (7.4)
Indigenous American/First Nations	1 (0.3)
Middle Eastern	10 (3.4)
South Asian	16 (5.4)
Southeast Asian	5 (1.7)
White	187 (63.0)
Prefer not to say	28 (9.4)
Not listed; please specify	1 (0.3)
Academic rank	
Assistant professor	172 (58.7)
Associate professor	121 (41.3)
Time as faculty physician, y	
<5	150 (50.0)
6-10	87 (29.0)
≥11	63 (21.0)

#### Attitudes Toward Mentorship

Table 2 describes the overall attitudes and preferences of junior surgical faculty related to mentorship. Nearly all survey participants (319 [95.8%]) strongly agreed/agreed that mentorship is important to their surgical career. The top areas where faculty agreed mentorship was important were professional networking (309 [92.8%]) and career advancement (301 [90.4%]), followed by research (294 [88.3%]), leadership skills (281 [84.4%]), and clinical skills (273 [82.0%]). Nearly two-thirds of the faculty agreed that emotional well-being (225 [67.6%]) and interpersonal skills (196 [58.9%]) were also important areas for mentorship (Table 2).

**Table 2.** Overall Attitudes and Preferences Related to Mentorship

Question	No. (%)			*P* value^a^
	Entire cohort	Male faculty	Female faculty	
Strongly agree or agree mentorship is important				
Research	294 (88.3)	138 (89.0)	119 (88.8)	.95
Career advancement	301 (90.4)	140 (90.3)	125 (93.3)	.36
Emotional well-being	225 (67.6)	102 (65.8)	96 (71.6)	.29
Professional networking	309 (92.8)	144 (92.9)	127 (94.8)	.51
Clinical skills	273 (82.0)	131 (84.5)	107 (79.9)	.30
Interpersonal skills	196 (58.9)	100 (64.5)	73 (54.5)	.08
Leadership skills	281 (84.4)	135 (87.1)	114 (85.1)	.62
Most valued characteristics in a mentor (choose 3)				
Integrity	147 (47.9)	82 (52.9)	56 (42.4)	.08
Dependability	110 (35.8)	58 (37.4)	45 (34.1)	.56
Flexibility	10 (3.3)	5 (3.2)	3 (2.3)	.63
Approachability	161 (52.4)	85 (54.8)	67 (50.8)	.49
Trustworthiness	137 (44.6)	69 (44.5)	60 (45.5)	.87
Inspiring	79 (25.7)	50 (32.3)	27 (20.5)	.02
Knowledgeable	170 (55.4)	82 (52.9)	76 (57.6)	.43
Respectful	64 (20.8)	35 (22.6)	23 (17.4)	.28
Skilled	89 (29.0)	51 (32.9)	31 (23.5)	.08
Other	15 (4.9)	6 (3.9)	7 (5.3)	.56
Gender preference of mentor				
Same gender as me	35 (11.6)	16 (10.3)	15 (11.4)	.63
Different gender as me	1 (0.3)	1 (0.6)	0	
Have no preference	271 (88.3)	138 (89.0)	117 (88.6)	
Race preference of mentor				
Same race as me	3 (1.0)	1 (0.6)	1 (0.8)	>.99
Different race as me	0	0	0	
Have no preference	304 (99.0)	154 (99.4)	131 (99.2)	
Comfort with requesting to change mentors				
Highly uncomfortable/uncomfortable	56 (18.9)	28 (18.7)	25 (19.4)	.32
Neither comfortable nor uncomfortable	133 (44.8)	63 (42.0)	64 (49.6)	
Highly comfortable/comfortable	108 (36.3)	59 (39.3)	40 (31.0)	
Have good mentor options if wished to change				
Yes	154 (52.2)	81 (54.0)	64 (50.0)	.51
No	141 (47.8)	69 (46.0)	64 (50.0)	

^a^ Comparing male vs female junior faculty.

#### Desirable Characteristics in a Mentor

When asked what characteristics faculty valued in a mentor, being knowledgeable (170 [55.4%]), approachable (161 [52.4%]), and having integrity (147 [47.9%]) were the most frequently selected while being flexible (10 [3.3%]) was the least frequently selected. When asked about their mentor preferences, 271 respondents (88.3%) and 304 participants (99.0%) reported no gender or race preference, respectively. These findings were consistent among male and female faculty (Table 2).

#### Changing Mentors

Survey participants were asked how comfortable they felt requesting to change their mentor. Of the respondents, 56 (19.0%) reported being highly uncomfortable/uncomfortable, 133 (44.8%) reported being neither comfortable nor uncomfortable, and 108 (36.3%) reported being highly comfortable/comfortable with requesting to change mentors. When asked if they have good mentor options if they wish to change, 141 respondents (47.8%) reported no (male, 69 [46%], vs female, 64 [50%]; *P* = .51) (Table 2).

#### Experiences of Formal vs Informal Mentorship

When we examined the formal vs informal mentorship experience as described in Table 3, only 58 faculty members (18.3%) surveyed had a formal mentor assigned compared with 261 (84.2%) who had at least 1 informal mentor (*P* < .001). Furthermore, 139 faculty members (53.3%) reported having 3 or more informal mentors. A higher proportion of those with informal mentors reported they were highly satisfied/satisfied with the mentorship provided compared with those with a formal mentor (221 [85.0%] vs 40 [69.0%]; *P* = .01) (Figure). Specifically, faculty were more highly satisfied/satisfied with the informal mentorship provided for clinical skills compared with formal mentorship (193 [74.2%] vs 33 [57.9%]; *P* = .01). When asked about which areas formal mentors provided the most and least helpful mentorship, faculty reported career advancement (32 [57.1%]) as the most helpful, and emotional well-being (18 [33.3%]) and interpersonal/leadership skills (18 [33.3%]) as the least helpful. In contrast, the most helpful area for informal mentorship was clinical skills (65 [25.9%), and the least helpful area was research (75 [30.4%) (Table 3).

**Table 3.** Experiences With Formal vs Informal Mentorship

Experience	Formal mentorship, No. (%)	Informal mentorship, No. (%)	*P* value
Have mentor(s)	58 (18.3)	261 (84.2)	<.001
Frequency of meetings with mentor			.37
Have not met with them	5 (8.6)	13 (5.0)	
Once a year	18 (31.0)	72 (27.8)	
Once a month	25 (43.1)	104 (40.2)	
Once a week or more	10 (17.2)	70 (27.0)	
Satisfaction with mentor			.01
Highly unsatisfied/unsatisfied	8 (13.8)	20 (7.7)	
Neither satisfied nor unsatisfied	10 (17.2)	19 (7.3)	
Highly satisfied/satisfied	40 (69.0)	221 (85.0)	
Highly satisfied or satisfied with mentorship provided			
Research	30 (52.6)	153 (58.8)	.39
Career advancement	44 (77.2)	210 (80.8)	.54
Emotional well-being	33 (57.9)	173 (66.5)	.22
Professional networking	39 (68.4)	195 (75.0)	.31
Clinical skills	33 (57.9)	193 (74.2)	.01
Interpersonal skills	32 (56.1)	157 (60.4)	.55
Leadership skills	34 (59.6)	184 (70.8)	.10
Most helpful area for mentorship			
Research	7 (12.5)	41 (16.3)	
Career advancement	32 (57.1)	62 (24.7)	
Emotional well-being	2 (3.6)	27 (10.8)	
Professional networking	5 (8.9)	28 (11.2)	
Clinical skills	8 (14.3)	65 (25.9)	
Interpersonal and leadership skills	2 (3.6)	28 (11.2)	
Least helpful area for mentorship			
Research	8 (14.8)	75 (30.4)	
Career advancement	3 (5.6)	20 (8.1)	
Emotional well-being	18 (33.3)	47 (19.0)	
Professional networking	1 (1.9)	22 (8.9)	
Clinical skills	6 (11.1)	40 (16.2)	
Interpersonal and leadership skills	18 (33.3)	43 (17.4)	

**Figure.**
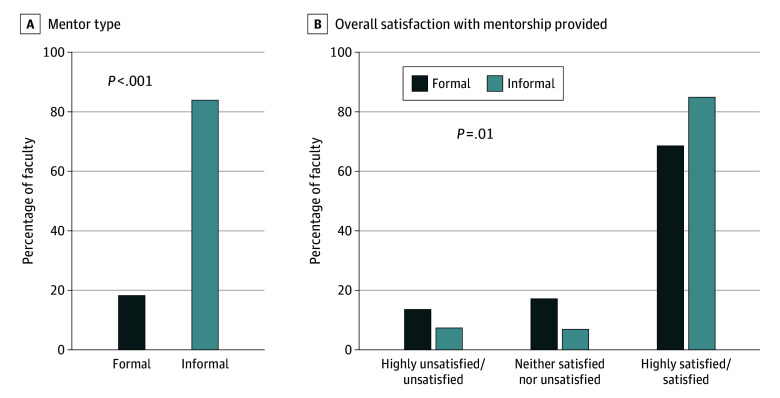
Formal vs Informal Mentorship Experience A. Proportion of junior surgical faculty with formal vs informal mentors. B. Satisfaction with mentorship.

#### Gender-Based Variations in Mentorship Experiences

On a subset analysis of survey results based on gender (Table 4), male and female junior faculty had similar rates of formal mentors (30 [19.6] vs 26 [19.4%], respectively; *P* = .97) and similar rates of satisfaction with their formal mentors (highly satisfied/satisfied, 20 [66.7%] male respondents vs 19 female [73.1%]; *P* = .10). However, male faculty with formal mentors were more highly satisfied/satisfied with the formal mentorship provided for emotional well-being (21 [70.0%] vs 11 [42.3%], respectively; *P* = .04) and interpersonal skills (21 [70.0%] vs 10 [38.5%]; *P* = .02) than female faculty. Male and female faculty agreed on the most helpful and least helpful areas of formal mentorship, except male faculty selected clinical skills as the most helpful area of formal mentorship more frequently than female faculty (7 [24.1%] vs 1 [4.0%], respectively; *P* = .04) (Table 4).

**Table 4.** Experiences With Formal and Informal Mentorship Among Male vs Female Junior Faculty

Experience	Formal mentorship			Informal mentorship		
	Male faculty, No. (%)	Female faculty, No. (%)	*P* value	Male faculty, No. (%)	Female faculty, No. (%)	*P* value
Have mentor(s)	30 (19.6)	26 (19.4)	.97	123 (79.4)	123 (91.8)	.003
Frequency of meetings with mentor			.05			.01
Have not met with them	3 (10.0)	2 (7.7)		6 (4.9)	7 (5.7)	
Once a year	9 (30.0)	8 (30.8)		36 (29.3)	31 (25.4)	
Once a month	9 (30.0)	15 (57.7)		38 (30.9)	60 (49.2)	
Once a week or more	9 (30.0)	1 (3.8)		43 (35.0)	24 (19.7)	
Satisfaction with mentor			.10			.60
Highly unsatisfied/unsatisfied	2 (6.7)	5 (19.2)		9 (7.4)	10 (8.1)	
Neither satisfied nor unsatisfied	8 (26.7)	2 (7.7)		7 (5.7)	11 (8.9)	
Highly satisfied/satisfied	20 (66.7)	19 (73.1)		106 (86.9)	102 (82.9)	
Highly satisfied or satisfied with mentorship provided						
Research	16 (53.3)	13 (50.0)	.80	71 (58.2)	76 (61.8)	.57
Career advancement	24 (80.0)	19 (73.1)	.54	102 (83.6)	96 (78.0)	.27
Emotional well-being	21 (70.0)	11 (42.3)	.04	82 (67.2)	83 (67.5)	.96
Professional networking	22 (73.3)	16 (61.5)	.35	93 (76.2)	90 (73.2)	.58
Clinical skills	20 (66.7)	12 (46.2)	.12	91 (74.6)	91 (74.0)	.91
Interpersonal skills	21 (70.0)	10 (38.5)	.02	75 (61.5)	74 (60.2)	.83
Leadership skills	20 (66.7)	13 (50.0)	.21	91 (74.6)	85 (69.1)	.34
Most helpful area for mentorship						
Research	2 (6.9)	5 (20.0)	.15	21 (17.5)	18 (15.5)	.68
Career advancement	16 (55.2)	16 (64.0)	.51	35 (29.2)	23 (19.8)	.10
Emotional well-being	1 (3.4)	0	NA	10 (8.3)	17 (14.7)	.13
Professional networking	1 (3.4)	3 (12.0)	.23	11 (9.2)	15 (12.9)	.36
Clinical skills	7 (24.1)	1 (4.0)	.04	32 (26.7)	29 (25.0)	.77
Interpersonal and leadership skills	2 (6.9)	0	NA	11 (9.2)	14 (12.1)	.47
Least helpful area for mentorship						
Research	5 (17.9)	2 (8.0)	.29	38 (33.0)	30 (25.6)	.22
Career advancement	1 (3.6)	2 (8.0)	.49	7 (6.1)	12 (10.3)	.25
Emotional well-being	8 (28.6)	10 (40.0)	.38	22 (19.1)	23 (19.7)	.92
Professional networking	1 (3.6)	0	NA	8 (7.0)	12 (10.3)	.37
Clinical skills	2 (7.1)	3 (12.0)	.55	18 (15.7)	21 (17.9)	.64
Interpersonal and leadership skills	11 (39.3)	8 (32.0)	.58	22 (19.1)	19 (16.2)	.56

Abbreviation: NA, not applicable.

In contrast, female faculty were more likely than male faculty to have at least 1 informal mentor (123 [91.8%] vs 123 [79.4%], respectively; *P* = .003) (Table 4), and more often had 3 or more informal mentors (78 [63.4%] vs 57 [46.3%], respectively; *P* = .007). Male faculty met with their informal mentors more often than female faculty (once a week or more, 43 [35.0%] vs 24 [19.7%], respectively; *P* = .01), but there were no significant differences in satisfaction with their informal mentors between the 2 groups. Career advancement (35 male [29.2%] vs 23 female faculty [19.8%]; *P* = .10) and clinical skills (32 male [26.7%] vs 29 female faculty [25.0%]; *P* = .77) were selected as the most helpful areas for informal mentorship, and research (38 male [33.0%] vs 30 female faculty [25.6%]; *P* = .22) the least helpful area for informal mentorship by both groups.

### Phase 2: Thematic Analysis of Semistructured Interviews

From the semistructured interviews, we identified 6 main themes: absence of mentorship infrastructure, mentee burden and responsibility, informal mentorship, mentor-mentee fit and relationship, preferred mentor characteristics, and optimizing mentorship (eAppendix 3 in Supplement 1).

Junior surgical faculty reiterated that most departments do not have a formal mentorship program for faculty. Instead, mentorship needs to be sought out by the mentee. As 1 junior faculty expressed, “I think the onus to find a mentor has been on me as a junior faculty” (eAppendix 3 in Supplement 1).

As for informal mentorship, most interview participants had multiple informal mentors for different areas of their career. One stated, “There are people who do different parts of mentorship for me.… I have … research mentors and clinical mentors.” Additionally, many mentees cited their senior partners as informal mentors: “As soon as I joined, the department chair and my senior partner have naturally become my mentors.” Junior faculty further expressed it is easier and more comfortable to form a relationship with an informal mentor rather than one that is assigned to you, with 1 person stating, “I think it’s easier to have more casual mentorship relationships with … your senior partners … than a formal thing that’s more burdensome” (eAppendix 3 in Supplement 1).

Furthermore, mentees often formed closer relationships to mentors who were similar to them. One female faculty said “When I find a female surgeon, it is easier for me to receive mentorship from that person.” Other interviewees stated they wanted mentors with similar backgrounds, such as those with children, who could help guide them in specific scenarios related to work-life integration. For example, 1 faculty said, “I still struggle with being a female with kids and surgery, and having a mentor in that space would be great” (eAppendix 3 in Supplement 1).

Other attributes of a good mentor highlighted in the interviews were the ability to listen and communicate based on such comments as, “As far as good attributes, being a good listener…” and “Most important is [the] ability or willingness to communicate.” Junior faculty also reported they would like more mentorship specifically in career advancement. “Understanding the academic process, what’s required for promotion, to understand the politics of academic medicine would be very helpful,” said 1 faculty (eAppendix 3 in Supplement 1).

## Discussion

To our knowledge, MAAPS is the largest US study to date that examines mentorship in academic surgery from the perspective of junior faculty. Consistent with the study by Kibbe et al,^16^ we found a strikingly low percentage of junior faculty had formal mentors (18.3%). In contrast, the majority of the junior faculty had informal mentors (84.2%), and more than half had 3 or more informal mentors. These findings indicate that faculty actively seek mentorship and recognize the need for multiple mentors to support the different dimensions of their career development. Mentee satisfaction with their formal mentors was significantly lower than with their informal mentors. This suggests that assigning mentors does not necessarily lead to successful mentor-mentee relationships. In a study by Phitayakorn et al^17^ of a mandatory mentorship pilot program in a surgical department, junior faculty reported the program assisted with career planning but did not help with work-life balance and overall job satisfaction. Another single-institution study examined the perspectives of junior faculty (n = 35) regarding their surgical mentorship program and identified more thoughtful mentor-mentee pairings as an area of improvement.^23^ Junior faculty in our study expressed similar disappointment about the lack of support departments provided in identifying the right mentors. Even more distressing is nearly 50% of survey participants felt they did not have good mentor options if they wanted to change mentors. These observations suggest a need to rethink the current approach to mentorship programs and make efforts to improve mentor-mentee pairings.

Furthermore, for those with informal mentors, mentees identified research as a deficient area for mentorship. This is an important finding because research and scholarly productivity are vital for career advancement in academia.^24^ Ideally, research mentors have expertise and are well connected in the field to serve as sponsors. A qualitative study by Highet et al^25^ also reported 12 strategies in 5 categories to improve mentorship of surgeon scientists, namely, initiating the relationship, identifying research focus, providing oversight, developing the research expertise of the mentee, and promoting self-sufficiency. Although research success is affected by multiple factors, pairing junior faculty with research mentors who have similar interests and can provide mentorship tailored to the junior faculty’s goals during their early careers could lead to better satisfaction with research mentorship.

Another significant finding from the survey results is that mentees do not have a race and ethnicity or gender preference for their mentors. This finding held true even when looking at male and female faculty results separately and is concordant with findings from previous reports.^24,26,27,28^ We acknowledge there are studies showing that gender concordant mentorship is important for specialty selection by trainees.^29,30,31^ Hence, there may be differences between faculty and trainee mentor preferences when it comes to gender. However, mentees still value mentors who have similar backgrounds as them, such as being parents. They also seek mentorship from their senior partners, as those mentors may better understand the clinical nuances faced by the mentee. Perhaps, gender and race are less important than similarities in professional or personal background when it comes to forming successful mentor-mentee relationships.

Based on the observations from our study, it is evident mentorship for academic junior surgical faculty requires significant attention and improvement. We propose a shift in the role of programs from assigning mentors to facilitating mentorship by pairing junior faculty with a mentorship guide or director, whose role would be to have in-depth discussions with the junior faculty to understand their career goals and identify suitable mentors. The role of mentorship guide/director could be fulfilled by division chiefs or chairs in some institutions. The junior faculty would then be responsible for initiating connections between potential mentors and developing mentor-mentee relationships. It is important the mentees drive this process because the success of mentor-mentee relationships is rooted in the organic development of connection and trust. Periodic meetings with junior faculty and the mentorship director to assess the trajectory of the faculty’s career development in the desired domains are also essential to pivot and provide further support. This framework takes the burden off the junior faculty to find mentors but still provides them the freedom to develop the relationship.

We recognize further work is needed to develop metrics of effective mentorship in the proposed approach. The adaptability of this approach at various institutions depends on the availability of suitable faculty who can serve as mentorship directors, as they should be well connected, be invested in the success of junior faculty, and have adequate time to be successful in this role. Needless to say, support from the institutions and department chairs and commitment from senior faculty are critical to the success of this approach. Investing in mentorship has a high probability to pay dividends in the success, growth, and retention of faculty and to help avert burnout.

### Limitations

This study has potential limitations. First, we did not have details regarding formal mentorship program structures. Second, we focused specifically on the views of mentees and not mentors. Additional insight from mentors regarding mentorship in academic surgery could help inform avenues for implementing more effective mentorship programs. Third, we did not examine career development outcomes, such as promotion, in relation to mentorship. Finally, most participants in our study self-identify as White. Future work should focus on the views of underrepresented groups in medicine, as more obstacles to accessing successful mentor-mentee relationships for these populations may be identified.

## Conclusions

Mentorship is vital for career advancement, but mentorship for junior surgical faculty needs more attention and active efforts for improvement. We found that formal mentorship programs in academic surgery are both less common and less effective in establishing strong mentor-mentee relationships than informal mentorship. Facilitating mentorship from the mentee perspective with the support and commitment from all stakeholders, including the institution, department, and mentors, is needed.

## Notes

Supplement 1. **eAppendix 1.** Survey questions **eAppendix 2.** Interview questions **eAppendix 3.** Themes from interviews and supporting excerpts

Supplement 2. Data sharing statement
