# Supplementary material for: Mentorship of Junior Surgical Faculty Across Academic Programs in Surgery
Source: JAMA Surg. 2024 Sep 4;159(11):1252–60. doi: 10.1001/jamasurg.2024.3390 (PMC11375519; doi:10.1001/jamasurg.2024.3390)
Supplement: Supplement 1. — eAppendix 1. Survey questions eAppendix 2. Interview questions eAppendix 3. Themes from interviews and supporting excerpts [file jamasurg-e243390-s001.pdf]

Supplemental Online Content

Yu J, Ruhi-Williams P, de Virgilio C, et al. Mentorship of junior surgical faculty across academic programs in surgery. *JAMA Surg*. Published online September 4, 2024. doi:10.1001/jamasurg.2024.3390

eAppendix 1. Survey questions

eAppendix 2. Interview questions

eAppendix 3. Themes from interviews and supporting excerpts

This supplemental material has been provided by the authors to give readers additional information about their work.

© 2024 Yu J et al. *JAMA Surgery.*

eAppendix 1: Survey questions

1	Mentorship is important for my surgical career.
2	Please reply to this statement for each category. Mentorship is important in my surgical career development with respect to: research, career advancement, emotional well-being, professional networking, clinical skills, interpersonal skills, leadership skills.
3	Do you have a formal mentor (someone assigned to you by your department) assigned to you?
4	How often do you meet with your formal mentor?
5	Please rate your overall satisfaction with the mentorship provided by your formal mentor.
6	Please rate your satisfaction with the mentorship provided by your formal mentor with respect to: research, career advancement, emotional well-being, professional networking, clinical skills, interpersonal skills, leadership skills.
7	In which of these areas do you feel that your formal mentor provides the most helpful mentorship? (Choose one)
8	In which of these areas do you feel that your formal mentor provides the least helpful mentorship? (Choose one)
9	Do you have an informal mentor (someone you seek out on your own)?
10	How many informal mentors do you have?
11	Where do the majority of your informal mentors work in relation to you?
12	How often do you meet with your informal mentor(s)?
13	Please rate your overall satisfaction with the mentorship provided by your informal mentor(s)
14	Please rate your satisfaction with the mentorship provided by your informal mentor(s) with respect to: research, career advancement, emotional well-being, professional networking, clinical skills, interpersonal skills, leadership skills.
15	In which of these areas do you feel that your informal mentor provides the most helpful mentorship? (Choose one)
16	In which of these areas do you feel that your informal mentor provides the least helpful mentorship? (Choose one)
17	What characteristics do you value most in a mentor? (Please choose up to 3)
18	What is your preference with respect to the gender of your mentor(s)?
19	What is your preference with respect to the race of your mentor(s)?
20	How comfortable would you feel requesting to change to another mentor?
21	If I wish to change mentors, I feel that I have other good mentor options.
22	What is your academic rank?
23	How long have you been a faculty physician?
24	What is your age?
25	What is your gender?
26	Please describe your race/ethnicity. (Check all that apply)

© 2024 Yu J et al. *JAMA Surgery.*

eAppendix 2: Interview questions

1	What are the skills of an effective mentor?
2	What attributes or characteristics do you find most important in a mentor? Why are those important to you?
3	Is “mentoring” considered normal within your work environment?
4	Are there any formal policies/rules/expectations about mentoring in your workplace?
5	How difficult or easy has it been to find mentorship in obtaining a mentor? What obstacles have you encountered in obtaining a mentor?
6	How frequently do you meet with your mentor?
7	Has mentorship played a significant role in your career to this point? If yes, what aspects of your career has mentorship benefited you? Specifically, has mentorship helped you with research?
8	Do you wish you had better mentorship in any area of your career? If yes, which areas?
9	If you wished to switch mentors, do you have options to switch?
10	Have you found that mentorship opportunities are equally available/accessible to everyone from different backgrounds (diversity and inclusion) in your department?

© 2024 Yu J et al. *JAMA Surgery.*

eAppendix 3: Themes from interviews and supporting excerpts

**Theme**	**Description and Illustrative Quotes**
**Absence of mentorship infrastructure**	Most institutions do not provide formal mentorship to faculty, although formal mentorship appears to be the norm for residents.<br><br>*"At our institution there is no formal mentorship process.*<br><br>*We have formal mentoring for residents but not for faculty."*
**Mentee burden and responsibility**	Mentorship is often something that needs to be sought out by the mentee, sometimes even outside of their institution.<br><br>*"I think the onus to find a mentor has been on me as a junior faculty."*
**Informal mentorship**	The majority of junior faculty have multiple informal mentors for different areas of their career. It is easier and more comfortable for them to form a relationship with an informal mentor rather than one that is assigned.<br><br>*"There are people who do different parts of mentorship for me… I have like research mentors and clinical mentors."*<br><br>*"I realized I needed a mentorship team."*<br><br>*"I think it's easier to have more casual mentorship relationships with like your senior partners… than a formal thing that's more burdensome."*
**Mentor-mentee fit and relationship**	Mentees often form closer relationships to mentors who are similar to them.<br><br>*"It's really easy to mentor people who you see similarities in. Like when I find a female surgeon, it's easier for me to receive mentorship from that person."*
**Preferred mentor characteristics**	Junior faculty want a mentor who is available, listens and communicates well.<br><br>*"…most important is ability or willingness to communicate."*<br><br>*"They should be available and easy to contact."*<br><br>*"As far as good attributes, being a good listener … "*
**Optimizing mentorship**	Junior faculty would like more mentorship specifically in career advancement.<br><br>*"Understanding the academic process, what's required for promotion, to understand the politics of academic medicine would be very helpful."*

© 2024 Yu J et al. *JAMA Surgery.*
