# Supplementary material for: Mentorship of Junior Surgical Faculty Across Academic Programs in Surgery
Source: JAMA Surg. 2024 Sep 4;159(11):1252–60. doi: 10.1001/jamasurg.2024.3390 (PMC11375519; doi:10.1001/jamasurg.2024.3390)
Supplement: Supplement 2. — Data sharing statement [file jamasurg-e243390-s002.pdf]

Data Sharing Statement

Yu. Mentorship of Junior Surgical Faculty Across Academic Programs in Surgery. *JAMA Surg*. Published September 04, 2024. doi:10.1001/jamasurg.2024.3390

Data
Data available: No

Additional Information
Explanation for why data not available: Our study does not include patient data. For requests for survey and interview data, please email Dr. Maheswari Senthil at maheswas@hs.uci.edu.
